# Nutritional status, abdominal muscle thickness, and functional outcomes in patients with subacute to chronic stroke

**DOI:** 10.3389/fneur.2026.1794367

**Published:** 2026-04-28

**Authors:** Esra Şahingöz Bakırcı, Yasin Coşkun, Tuğba Alışık

**Affiliations:** 1 Department of Physical Medicine and Rehabilitation, Faculty of Medicine, Yozgat Bozok University, Yozgat, Türkiye; 2 Department of Physical Medicine and Rehabilitation, AIBU Izzet Baysal Physical Treatment and Rehabilitation Training and Research Hospital, Bolu, Türkiye

**Keywords:** abdominal muscles, mini nutritional assessment, nutritional status, Prognostic Nutritional Index, stroke rehabilitation, ultrasonography

## Abstract

**Background:**

Nutritional status is an important determinant of rehabilitation outcomes after stroke; however, its relationship with abdominal muscle morphology remains unclear. Given the role of abdominal muscles in trunk stability, postural control, and functional mobility, this study aimed to evaluate the association between nutritional status, abdominal muscle thickness, and functional recovery in patients with subacute to chronic stroke.

**Methods:**

In this prospective observational study, patients with subacute to chronic stroke who were admitted to an inpatient rehabilitation program were evaluated at baseline and after a 4-week rehabilitation program. Nutritional status was assessed using the Mini Nutritional Assessment–Short Form (MNA-SF), reflecting screening-defined nutritional risk. Abdominal muscle thicknesses of the external oblique, internal oblique, and transversus abdominis were measured by ultrasonography on the non-paretic side. Clinical, functional, cognitive, and biochemical parameters were recorded at baseline and discharge. The primary outcome was functional independence at discharge, assessed by the Barthel Index.

**Results:**

Sixty patients were included, of whom 38 had normal nutritional status and 22 were classified as having nutritional risk based on the MNA-SF. Significant within-group improvements in motor recovery and mobility were observed in both groups following rehabilitation (*p* < 0.05). Although both groups demonstrated improvements, patients with normal nutritional status had higher functional independence levels at both baseline and post-treatment assessments. Baseline abdominal muscle thicknesses were similar between groups (*p* > 0.05) and did not change significantly after rehabilitation (*p* > 0.05). Multivariable analysis showed that nutritional risk was independently associated with lower discharge Barthel Index scores (B = −2.99, 95% CI −5.65 to −0.33, *p* = 0.028), after adjustment for prespecified covariates. In contrast, nutritional risk was not independently associated with discharge cognitive outcomes (MMSE) or mobility outcomes (FAC and mRS).

**Conclusion:**

In patients with subacute to chronic stroke, functional recovery during rehabilitation appears to be associated with nutritional risk, whereas no significant association was observed with abdominal muscle thickness. Although muscle thickness remained unchanged, screening-defined nutritional risk was independently associated with functional independence at discharge. These findings underscore the importance of early nutritional screening and comprehensive evaluation during stroke rehabilitation.

## Introduction

Stroke is the second leading cause of death worldwide and the third leading cause of disability-adjusted life years (DALYs) lost due to death and disability, thereby representing a substantial global health burden (1, 2). A large proportion of stroke survivors experience varying degrees of impaired independent mobility and functional limitations, making rehabilitation an indispensable component of stroke management (3, 4).

Malnutrition is a common clinical challenge across all phases of stroke care, with prevalence rates ranging from 6.1 to 62% due to neurological and cognitive impairments (5, 6). While acute phase nutrition has been extensively studied, the impact of nutritional status on clinical outcomes during the chronic phase remains relatively overlooked (7). This status has a direct, negative impact on overall survival and rehabilitation; malnutrition is associated with increased complications, prolonged hospital stays, and higher mortality (5, 8, 9). Factors such as pre-existing malnutrition, dysphagia, prior stroke history, diabetes mellitus, immobilization, and reduced level of consciousness have been identified as being associated with an increased risk of undernutrition in patients with stroke (10). Poor nutritional status may adversely affect post-stroke recovery and rehabilitation by contributing to muscle loss and the development of sarcopenia (11). Improvements in nutritional status and diet quality have been shown to positively influence motor and cognitive functions, handgrip strength, and quality of life, thereby contributing to reduced disability in stroke patients (12–14). In a multicenter retrospective cohort study, approximately one in nine patients with severe stroke admitted to a rehabilitation unit during the subacute phase was found to be malnourished, which was associated with poor functional outcomes (15). Given the negative prognostic impact of malnutrition in stroke, early nutritional assessment and appropriate interventions are essential. A multidisciplinary approach that includes continuous monitoring of nutritional status, timely nutritional support, and dietary modifications beyond hospital discharge is therefore critical (12).

A wide range of methods are currently used to assess nutritional status in patients with stroke, including laboratory parameters, anthropometric measurements, and screening tools (16). The Global Leadership Initiative on Malnutrition (GLIM) criteria are widely accepted as an international framework for diagnosing malnutrition. They combine phenotypic findings such as weight loss, low body mass index, and reduced muscle mass with etiologic factors like reduced food intake or inflammation. However, in stroke patients, assessing muscle mass can be challenging because neurogenic atrophy and nutrition related sarcopenia may overlap (17). The Mini Nutritional Assessment–Short Form (MNA-SF) is one of the most commonly used nutritional screening tools in stroke patients and has been shown to be associated with various functional outcomes (18). The Prognostic Nutritional Index (PNI), calculated based on serum albumin levels and lymphocyte counts, is a laboratory based nutritional assessment tool that has been previously applied in stroke populations, with different cutoff values proposed across studies (19–21).

In recent years, ultrasonography (US) has emerged as a non-invasive, practical, and reproducible modality for evaluating muscle atrophy following a stroke. To date, investigations into post-stroke muscle morphology have predominantly focused on the lower extremities; for instance, alterations in quadriceps thickness have been closely linked to nutritional intake and the timing of gait initiation (22–24). Despite their clinical significance, abdominal muscles, which are fundamental to trunk stability, postural control, and functional mobility, have received relatively limited attention in the stroke population. Furthermore, existing research on trunk muscle thickness often relies solely on structural assessments, frequently overlooking systemic factors like nutritional status that may significantly influence muscle characteristics (25–28). The success of post-stroke rehabilitation is fundamentally tied to a patient’s ability to regain trunk control and achieve independent mobility. While some evidence suggests a link between abdominal muscle thickness and functional capacity, the exact structural evolution of these muscles after a stroke is not yet fully understood (29). Interestingly, abdominal muscles receive bilateral neural input, which may grant them a degree of resilience against the structural loss typically seen in extremity muscles (27). Despite this potential advantage, how nutritional risk influences this relatively preserved muscle structure remains unclear. Although nutritional status is a cornerstone of muscle metabolism and systemic recovery, research has traditionally treated nutrition and muscle morphology as separate issues. Consequently, it is still difficult to discern whether functional gains during rehabilitation stem primarily from physical changes in the muscle or from neurophysiological adaptations. To address this gap, this study focuses on the relationship between nutritional status, abdominal muscle morphology, and functional recovery in patients with subacute to chronic stroke.

In this study, we evaluated nutritional risk using the MNA-SF together with ultrasonographic abdominal muscle thickness and laboratory markers, including serum albumin and the PNI, to better understand their combined relationship with functional recovery after stroke. We focused on patients with subacute to chronic stroke and explored whether nutritional status is independently associated with functional outcomes beyond measurable changes in muscle thickness. To our knowledge, this is the first longitudinal study to explore the interaction between these specific domains within a clinical stroke rehabilitation setting.

## Materials and methods

This prospective observational study was conducted at the Department of Physical Medicine and Rehabilitation of Abant İzzet Baysal University İzzet Baysal Physical Therapy and Rehabilitation Training and Research Hospital. Ethical approval was obtained from the Bolu Abant İzzet Baysal University Non-Interventional Clinical Research Ethics Committee (date: May 6, 2025; approval no: 2025/24). All procedures were carried out in accordance with the principles of the Declaration of Helsinki.

Adult patients with a diagnosis of stroke who voluntarily participated in an inpatient neurological rehabilitation program in the Physical Medicine and Rehabilitation clinic between June and September 2025 were included in the study. Written and verbal informed consent was obtained from all participants. Patients with central nervous system disorders other than stroke, a history of malignancy, severe infection, advanced organ failure, known eating disorders, no oral food intake, inflammatory bowel disease, short bowel syndrome, malabsorption, or cognitive impairment that could interfere with the completion of assessments were excluded from the study.

### Rehabilitation protocol

All participants were enrolled in a standardized 4-week inpatient rehabilitation program, consisting of 20 sessions delivered five days per week, with each session lasting approximately 60 min. The program was administered by a consistent team of licensed physiotherapists and followed the routine clinical protocols established at our center. At the core of the intervention was the enhancement of trunk stability and core activation. This involved mat-based exercises specifically designed to engage the abdominal musculature, including the internal and external obliques and the transversus abdominis, through pelvic tilts, bridging, and rhythmic stabilization in both seated and quadruped positions. For the upper limbs, the protocol prioritized motor control on the affected side by incorporating shoulder stabilization, active-assisted movements, and task-specific reaching and grasping exercises. Lower limb rehabilitation focused on progressive resistance training, where the intensity was carefully titrated using body weight and elastic bands according to each patient’s tolerance. Balance and functional mobility were addressed through a combination of static and dynamic postural control, weight-shifting, and sit-to-stand transitions, always emphasizing active trunk engagement. Gait training followed a task-oriented framework. To ensure consistency, functional outcomes were assessed by the same assessor at two time points: at baseline before the 4-week program (t0) and immediately upon its completion (t1).

Although the rehabilitation program was standardized in duration and content, the actual intensity likely varied across individuals depending on clinical status and tolerance. As objective measures of rehabilitation dose were not recorded, we were unable to compare therapy intensity between groups; thus, variability in treatment engagement may have influenced the outcomes and should be considered a potential source of residual confounding.

### Clinical assessment

Sociodemographic data, including age, sex, and body mass index (BMI), as well as clinical characteristics such as stroke type (ischemic/hemorrhagic), stroke duration, affected side, and accompanying comorbidities, were obtained from patients’ medical records.

Motor function was evaluated using the Brunnstrom Motor Recovery Staging system, which defines flaccid paralysis with no voluntary movement as Stage 1 and the ability to perform isolated joint movements as Stage 6 (30).

Ambulation ability was assessed using the Functional Ambulation Category (FAC), which consists of six categories scored from 0 to 5. A score of FAC-0 indicates the absence of independent ambulation, whereas increasing scores reflect greater levels of walking independence; FAC-5 represents independent ambulation on level surfaces (31).

Muscle tone was evaluated using the Modified Ashworth Scale (MAS). During assessment, joints were passively moved in a repetitive and rapid manner, and resistance to movement was graded accordingly. A score of 0 indicates no increase in muscle tone, while higher scores reflect increasing spasticity; a score of 4 denotes rigidity in flexion and extension (32).

The Barthel Index was used to assess the level of dependence in activities of daily living. The index consists of 10 items, including feeding, wheelchair–bed transfers, personal hygiene, toileting, bathing, walking on level surfaces, stair climbing, dressing, and bowel and bladder control. Total scores range from 0 to 100, with scores of 0–20 indicating total dependence, 21–61 severe dependence, 62–90 moderate dependence, 91–99 mild dependence, and 100 complete independence (33). The Modified Rankin Scale (mRS) was used to evaluate functional status and the degree of independence after stroke. The scale ranges from 0 to 6, where a score of 0 indicates no symptoms and a score of 6 represents death (34).

Cognitive status was assessed using the Mini-Mental State Examination (MMSE), which consists of 11 items evaluating orientation, attention, memory, language, calculation, and visuoconstructive abilities. Total scores range from 0 to 30 (35).

Nutritional status was assessed using the MNA-SF, a screening tool developed for the rapid and reliable identification of individuals at nutritional risk. Total scores range from 0 to 14, and participants were classified as having normal nutritional status (12–14 points) or being at nutritional risk (≤11 points) based on MNA-SF (36, 37). Accordingly, the exposure in this study reflects screening-defined nutritional risk rather than a clinical diagnosis of malnutrition.

All scales were administered twice: at hospital admission and after completion of the 4-week rehabilitation program.

### Laboratory assessments

From routine blood tests obtained at hospital admission and discharge, complete blood count parameters, C-reactive protein (CRP), albumin, ferritin, and total protein levels were recorded.

The Prognostic Nutritional Index (PNI) was calculated using the formula: 10 × serum albumin (g/dL) + 0.005 × peripheral lymphocyte count (/mm^3^) (38). Lower PNI values indicate an increased risk of malnutrition (39). Since serum albumin levels were recorded in g/L, albumin values were converted to g/dL by dividing by 10 before PNI calculation.

### Ultrasonographic evaluation

Abdominal muscle thickness, including the transversus abdominis, internal oblique, and external oblique muscles, was assessed using a high-frequency (7–15 MHz) linear transducer with an ultrasound device (V8 machine, Samsung Medison, Seoul, Korea) by a clinician with 5 years of experience in musculoskeletal ultrasonography. During the assessment, participants were positioned supine with hip joints flexed to 45 degrees. Measurements of the transversus abdominis, internal oblique, and external oblique muscles were performed 2.5 cm lateral to the umbilical midline at the midpoint between the lower costal margin and the iliac crest. Each measurement was repeated three times, and the mean value was recorded. To minimize measurement bias related to increased muscle tone and spasticity, ultrasonographic assessments were performed on the non-paretic side in all patients.

All ultrasonographic assessments were conducted at baseline and after completion of the 4-week rehabilitation program as post-treatment evaluations.

The primary outcome of the study was functional independence at discharge, assessed using the Barthel Index. Secondary outcomes included MMSE, FAC, mRS, abdominal muscle thickness, and laboratory parameters.

### Statistical analysis

Statistical analyses were performed using JASP 0.95.4 (JASP Team, University of Amsterdam, Amsterdam, The Netherlands). The distribution of continuous variables was assessed using the Shapiro–Wilk test and visual inspection of histograms. Normally distributed continuous variables were summarized as mean ± standard deviation, whereas non-normally distributed continuous variables were presented as median (interquartile range [IQR]: 25th–75th percentile). Categorical variables were expressed as number (percentage).

Baseline characteristics were compared according to nutritional risk group. To quantify baseline imbalance in this observational study, standardized mean differences (SMDs) were calculated for demographic, clinical, functional, cognitive, ultrasound, and laboratory variables, with group comparison *p* values provided for descriptive purposes. For comparisons between two independent groups, the independent-samples t test was used for normally distributed continuous variables, the Mann–Whitney U test for non-normally distributed continuous or ordinal variables, and the chi-square test or Fisher’s exact test, as appropriate, for categorical variables.

For within-group comparisons between baseline and post-treatment assessments, the paired-samples t test was used for normally distributed continuous variables and the Wilcoxon signed-rank test for non-normally distributed continuous variables and ordinal outcomes. Detailed response patterns in ordinal outcomes were additionally summarized using shift analyses, and between-group comparisons of improvement categories were performed using the chi-square test or Fisher’s exact test, as appropriate. For binary response outcomes, odds ratios (ORs) with 95% confidence intervals (CIs) were calculated. For between-group comparisons of change scores in non-normally distributed continuous variables, rank-biserial correlations with 95% CIs were reported as effect-size measures.

For the Barthel Index, clinically meaningful improvement was defined *a priori* using a threshold corresponding to the published minimal clinically important difference (MCID) in stroke populations. Hsieh et al. (40) estimated the MCID as 1.85 points on the 20-point Barthel Index, which corresponds approximately to 9.25 points on a 0–100 scale; accordingly, an increase of ≥9.8 points was used as the threshold for clinically meaningful improvement in the present study. For the MMSE, because a stroke-specific MCID is not well established, a > 1-point increase was examined as an exploratory indicator of cognitive improvement rather than as a formal MCID. The decision to examine clinically meaningful change in MMSE was informed by published literature on meaningful change thresholds in cognition-focused outcome research (41), while acknowledging that these thresholds are not stroke-specific.

To address confounding, multivariable regression analyses were performed using an ANCOVA-based approach for continuous outcomes, adjusting for prespecified baseline covariates including age, sex, time from stroke onset to rehabilitation, baseline outcome values, and clinically relevant stroke-related variables. To examine whether nutritional risk was independently associated with discharge functional outcome, multivariable linear regression was performed with discharge Barthel Index as the primary dependent variable. The primary adjusted model included nutritional risk group, baseline Barthel Index, age, sex, time from stroke onset to rehabilitation, stroke type, and baseline MMSE. A sensitivity model additionally included hypertension and baseline serum albumin. As secondary analyses, multivariable linear regression was also performed for discharge MMSE, and binary logistic regression was used to evaluate improvement in FAC and mRS. Logistic regression models included nutritional risk group, baseline scale value, age, sex, time from stroke onset to rehabilitation, and baseline MMSE. Stroke type was initially considered during model building; however, it was excluded from the final multivariable logistic regression models due to the limited number of hemorrhagic stroke cases (*n* = 6), which resulted in sparse data and unstable estimates consistent with quasi-complete separation.

Regression assumptions were evaluated using variance inflation factors, Durbin–Watson statistics for linear models, and influential case diagnostics. Because the analyses of MMSE, FAC, mRS, ultrasound, and laboratory change variables were considered secondary or exploratory, these findings were interpreted cautiously. All statistical tests were two-sided, and *p* < 0.05 was considered statistically significant.

There were no missing data for the variables included in the analyses; therefore, all analyses were conducted using a complete-case approach.

## Results

A total of 60 patients were included in the study, including 38 patients with normal nutritional status and 22 patients classified as having nutritional risk according to the MNA-SF at baseline. Baseline comparisons showed that the groups were similar across several demographic and stroke-related variables, including age, sex distribution, BMI, stroke type, affected side, time from stroke onset to rehabilitation, and most clinical complication variables. However, clinically relevant between group imbalances were observed for several variables, particularly hypertension, baseline functional status, cognitive status, and nutritional biomarkers. The largest standardized mean differences were observed for baseline Barthel Index, MMSE, serum albumin, and PNI, indicating worse baseline functional and nutritional status in patients with nutritional risk (Tables 1–3; Supplementary Figure 1).

**Table 1:** Baseline demographic, clinical, and stroke-related characteristics according to nutritional risk group.

Parameters	Subcategory/level	Patients with normal nutritional status	Patients with nutritional risk	SMD	Effect size	*p* value
Age (years)		67 (58.3–72)	64.5 (60.3–73.5)	−0.002	0.005 (−0.292 to 0.301)	0.982
Female Sex	Female	13 (34.2%)	8 (36.4%)	−0.045	0.022	0.866
Body mass index (kg/m^2^)		25.1 (22.6–29.5)	28.4 (22.2–31.3)	−0.253	−0.146 (−0.424 to 0.157)	0.353
Comorbidities and stroke-related characteristics						
Hypertension		20 (52.6%)	18 (81.8%)	−0.654	0.292	0.029
Diabetes mellitus		15 (39.5%)	4 (18.2%)	0.484	0.221	0.149
Coronary artery disease		4 (10.5%)	4 (18.2%)	−0.22	0.109	0.449
Thyroid disease		1 (2.6%)	2 (9.1%)	−0.278	0.143	0.548
Stroke type	Ischemic	34 (89.5%)	20 (90.9%)	−0.048	0.023	>0.999
	Hemorrhagic	4 (10.5%)	2 (9.1%)	0.048		
Affected side	Right	24 (63.2%)	10 (45.5%)	0.361	0.213	0.255
	Left	13 (34.2%)	12 (54.5%)	−0.418		
	Both side	1 (2.6%)	0 (0%)	0.232		
Time from stroke to rehabilitation (months)		2.5 (1.1–3)	2.3 (1.1–3)	0.31	0.1 (−0.202 to 0.385)	0.519
Clinical characteristics and complications						
Speech impairment		6 (15.8%)	4 (18.2%)	−0.064	0.031	0.811
Swallowing disorder	Absent	35 (92.1%)	22 (100%)	−0.414	0.175	0.401
	with solid foods	2 (5.3%)	0 (0%)	0.333		
	with liquid foods	1 (2.6%)	0 (0%)	0.232		
Reduced oral intake		10 (26.3%)	11 (50%)	−0.438	0.239	0.064
Unintentional weight loss		4 (10.5%)	6 (27.3%)	−0.438	0.217	0.149
Infection during hospitalization		19 (50%)	15 (68.2%)	0.376	0.177	0.171

SMD, standardized mean difference. *p* values were calculated using the Mann–Whitney U test, chi-square test as appropriate. Standardized mean differences (SMDs) are presented to quantify baseline imbalance. Effect sizes shown as Rank-biserial correlation for continuous variables and phi coefficient or Cramer’s V for categorical variables, where applicable.

At baseline, patients with nutritional risk tended to have lower Barthel Index scores (*p* = 0.054) and had significantly lower MMSE scores (*p* = 0.015). In contrast, abdominal muscle thickness measurements were largely similar between groups, with only modest imbalance in external oblique thickness (SMD: 0.251) and minimal imbalance for internal oblique and transversus abdominis thickness (SMD: 0.067 and −0.087, respectively). Laboratory comparisons further showed lower serum albumin and PNI values in patients with nutritional risk (*p* = 0.006 and *p* = 0.05, respectively), whereas most inflammatory and hematologic parameters did not differ significantly between groups (Tables 2, 3).

**Table 2:** Baseline functional, cognitive, and abdominal muscle measures according to nutritional risk group.

Parameters	Subcategory/level	Patients with normal nutritional status	Patients with nutritional risk	SMD	Effect size	*p* value
BMRS – Upper extremity						
	1	2 (5.3%)	1 (4.5%)	0.033	0.436	0.044
	2	3 (7.9%)	3 (13.6%)	−0.186		
	3	8 (21.1%)	10 (45.5%)	−0.536		
	4	7 (18.4%)	6 (27.3%)	−0.212		
	5	17 (44.7%)	1 (4.5%)	1.054		
	6	1 (2.6%)	1 (4.5%)	−0.103		
BMRS - Hand						
	1	4 (10.5%)	3 (13.6%)	−0.096	0.381	0.122
	2	4 (10.5%)	5 (22.7%)	−0.332		
	3	6 (15.8%)	6 (27.3%)	−0.282		
	4	9 (23.7%)	5 (22.7%)	0.023		
	5	15 (39.5%)	2 (9.1%)	0.758		
	6	0 (0%)	1 (4.5%)	−0.309		
BMRS – Lower extremity						
	1	N/A	N/A		0.418	0.033
	2	1 (2.6%)	0 (0%)	0.232		
	3	5 (13.2%)	9 (40.9%)	−0.658		
	4	11 (28.9%)	9 (40.9%)	−0.253		
	5	20 (52.6%)	4 (18.2%)	0.772		
	6	1 (2.6%)	0 (0%)	0.232		
FAC						
	0	0 (0%)	2 (9.1%)	−0.447	0.312	0.322
	1	7 (18.4%)	5 (22.7%)	−0.107		
	2	9 (23.7%)	5 (22.7%)	0.023		
	3	1 (2.6%)	2 (9.1%)	−0.278		
	4	12 (31.6%)	5 (22.7%)	0.2		
	5	9 (23.7%)	3 (13.6%)	0.26		
mRS						
	0	2 (5.3%)	0 (0%)	0.333	0.315	0.31
	1	16 (42.1%)	5 (22.7%)	0.423		
	2	2 (5.3%)	3 (13.6%)	−0.289		
	3	5 (13.2%)	4 (18.2%)	−0.139		
	4	13 (34.2%)	9 (40.9%)	−0.139		
	5	0 (0%)	1 (4.5%)	−0.309		
Barthel Index		82.5 (65–90)	65 (51.3–85)	0.608	0.3 (0.004 to 0.548)	0.054
MMSE		25 (22.5–26)	22 (22–24.8)	0.542	0.374 (0.088 to 0.604)	0.016
EO muscle thickness (cm)		3.4 (2.9–4.4)	3.5 (2.8–4.6)	0.251	0.094 (−0.208 to 0.38)	0.550
IO muscle thickness (cm)		5.4 (4.5–6.7)	5.6 (4.4–6.7)	0.067	0.031 (−0.268 to 0.325)	0.848
TrA muscle thickness (cm)		3.3 (2.8–4.4)	3.1 (2.7–4.3)	−0.087	0.059 (−0.242 to 0.349)	0.713

SMD, standard mean difference; BMRS, Brunnstrom Motor Recovery Stages; FAC, Functional Ambulation Category; mRS, Modified Rankin Scale; MMSE, Mini-Mental State Examination; EO, External oblique; IO, Internal oblique; TrA, Transversus abdominis. *p* values were calculated using the Mann–Whitney U test, chi-square test as appropriate. SMDs are presented to quantify baseline imbalance. Data shown as frequencies and percentages for categorical variables and median (interquartile range) for continuous variables. Effect sizes shown as Rank-biserial correlation for continuous variables and Cramer’s V for categorical variables, where applicable.

**Table 3:** Baseline laboratory and nutritional parameters according to nutritional risk group.

Parameters	Patients with normal nutritional status	Patients with nutritional risk	SMD	Effect size	*p* value
White blood cell count (10^3^/μL)	7.4 (6.5–8.9)	8.1 (6.9–8.8)	−0.156	−0.123 (−0.405 to 0.18)	0.434
Platelet count (10^3^/μL)	257 (209–300)	235.5 (186.8–317.8)	0.226	0.191 (−0.111 to 0.462)	0.223
Neutrophil count (10^3^/μL)	4.8 (3.7–5.2)	5 (4.2–5.9)	−0.28	−0.215 (−0.481 to 0.087)	0.170
Lymphocyte count (10^3^/μL)	2.1 (1.6–2.7)	2 (1.6–2.6)	0.13	0.093 (−0.209 to 0.379)	0.555
C-reactive protein (mg/L)	4.3 (2–7.8)	3.8 (2–6.8)	0.098	0.085 (−0.217 to 0.372)	0.585
Serum albumin (g/L)	44.7 (42.1–45.9)	41.3 (40–44.1)	0.719	0.433 (0.157 to 0.647)	0.006
Serum ferritin (ng/mL)	68.7 (36.4–116.6)	90 (36.5–171)	−0.321	−0.156 (−0.432 to 0.148)	0.322
Total protein (g/L)	69.5 (65.3–74.9)	69.7 (66–72.8)	0.196	0.019 (−0.279 to 0.314)	0.908
Creatinine (mg/dL)	0.8 (0.7–0.93)	0.8 (0.6–0.9)	0.32	0.105 (−0.197 to 0.39)	0.496
Prognostic Nutritional Index	54.2 (50.6–58.9)	51 (47.7–55.8)	0.542	0.306 (0.011 to 0.553)	0.050

SMD, standard mean difference. *p* values were calculated using the Mann–Whitney U test. SMDs are presented to quantify baseline imbalance. Effect sizes shown as Rank-biserial correlation for continuous variables.

Within-group analyses demonstrated significant pre- to post-treatment improvement in several clinical outcomes. In patients with normal nutritional status, significant within-group improvements were observed in Brunnstrom upper extremity, hand, and lower extremity stages, FAC, mRS, Barthel Index, and MMSE (all *p* < 0.05; Supplementary Table 1). In patients with nutritional risk, significant within-group improvements were observed in Brunnstrom upper extremity, hand, and lower extremity stages and FAC (all *p* < 0.05; Supplementary Table 1), whereas changes in mRS, Barthel Index, and MMSE were smaller and did not consistently reach statistical significance (all *p* > 0.05). Detailed shift analyses showed that the most common response pattern in both groups was either no change or a one-stage/category improvement, whereas larger category shifts were less frequent (Table 4; Supplementary Table 1).

**Table 4:** Clinical response outcomes after rehabilitation according to nutritional risk group.

Variables	Subcategory	Patients with normal nutritional status	Patients with nutritional risk	*p* value	OR (95% CI)
BMRS – Upper extremity	Unchanged	12 (31.6%)	8 (36.4%)	0.780	0.808 (0.267 to 2.440)
	≥1-stage improvement	26 (68.4%)	14 (63.6%)		
BMRS - Hand	Unchanged	7 (18.4%)	7 (31.8%)	0.237	0.484 (0.143 to 1.632)
	≥1-stage improvement	31 (81.6%)	15 (68.2%)		
BMRS – Lower extremity	Unchanged	14 (36.8%)	10 (45.5%)	0.512	0.700 (0.241 to 2.035)
	≥1-stage improvement	24 (63.2%)	12 (54.5%)		
FAC	Not-improved	27 (71.1%)	16 (72.7%)	0.890	0.920 (0.285 to 2.969)
	Improved	11 (28.9%)	6 (27.3%)		
mRS	Not-improved	27 (71.1%)	15 (68.2%)	0.815	1.145 (0.367 to 3.577)
	Improved	11 (28.9%)	7 (31.8%)		
Barthel Index	>MCID (9.8 point)	8 (21.1%)	4 (18.2%)	0.789	0.833 (0.219 to 3.166)
MMSE	>1-point increase	18 (47.4%)	7 (31.8%)	0.239	0.519 (0.173 to 1.558)
		Md (IQR)	Md (IQR)	*p* value	RBC (95% CI)
EO muscle thickness (cm)		−0.15 (−0.273 to 0.22)	−0.285 (−0.428 to 0.308)	0.514	−0.103 (−0.388 to 0.2)
IO muscle thickness (cm)		−0.32 (−0.595 to 0.22)	−0.19 (−0.61 to 0.435)	0.570	0.09 (−0.212 to 0.376)
TrA muscle thickness (cm)		−0.145 (−0.4 to 0.392)	−0.135 (−0.3 to 0.358)	0.586	0.086 (−0.216 to 0.373)
WBC (10^3^/μL)		−0.485 (−1.75 to 0.8)	−0.325 (−1.72 to 0.953)	0.597	0.084 (−0.218 to 0.371)
Platelet count (10^3^/μL)		3.5 (−20.5 to 11)	−21.5 (−40 to 1.25)	0.041	−0.319 (−0.563 to −0.025)
Neutrophil count (10^3^/μL)		−0.51 (−1.215 to 0.433)	−0.435 (−1.68 to 0.988)	0.890	0.023 (−0.276 to 0.317)
Lymphocyte count (10^3^/μL)		−0.015 (−0.253 to 0.293)	0.105 (−0.135 to 0.468)	0.076	0.278 (−0.021 to 0.53)
CRP (mg/L)		0 (−2.775 to 2.3)	0.05 (−1.625 to 3)	0.618	0.079 (−0.223 to 0.367)
Serum albumin (g/L)		−3.1 (−5.175 to −1)	−0.85 (−2.7 to 1.725)	0.005	0.438 (0.162 to 0.65)
Serum ferritin (ng/mL)		0.345 (−12.25 to 7.848)	−17.13 (−59.33 to −0.818)	0.009	−0.409 (−0.629 to −0.128)
Total protein (g/L)		−2.2 (−5.025 to 1.725)	−2.15 (−4.4 to 4.15)	0.759	0.049 (−0.251 to 0.341)
PNI		−3.7 (−5.312 to −0.262)	0.425 (−1.175 to 4.25)	< 0.001	0.561 (0.317 to 0.735)

SMD, standard mean difference; BMRS, Brunnstrom Motor Recovery Stages; FAC, Functional Ambulation Category; mRS, Modified Rankin Scale; MMSE, Mini-Mental State Examination; EO, External oblique; IO, Internal oblique; TrA, Transversus abdominis; WBC, White blood cell; CRP, C-reactive protein; PNI, Prognostic nutritional index; RBC, Rank biserial correlation; Md (IQR), Median (inter quartile range); >MCID (9.8 point), Over Minimally Clinically Important Difference in Barthel Index (≥9.8-point increase). Shift categories are presented descriptively as frequencies and percentages. Between-group comparisons of continuous values were performed using the Mann–Whitney U test and Effect sizes are reported as rank-biserial correlations with 95% confidence intervals, and between-group comparisons of shift distributions were assessed using the chi-square test and effect sizes are presented as odds ratios (ORs) with 95% confidence intervals (CIs).

Despite these within-group improvements, between-group comparisons of categorical clinical response did not show statistically significant differences for Brunnstrom upper extremity, hand, or lower extremity stages, FAC, mRS, clinically meaningful improvement in Barthel Index, or MMSE improvement of more than 1 point. Although the proportions generally favored patients with normal nutritional status for several outcomes, these differences were modest and accompanied by wide confidence intervals (Table 4).

No consistent between-group differences were observed in abdominal muscle thickness changes during rehabilitation. Similarly, most hematologic and biochemical parameters did not differ significantly between groups in terms of change over time. However, exploratory analyses suggested some heterogeneity in selected laboratory measures. In particular, platelet count change differed significantly between groups (*p* = 0.041), and rank-biserial effect sizes indicated relatively larger between-group differences for serum albumin, ferritin, and PNI change. Within-group reductions in serum albumin (*p* < 0.001) and PNI (*p* < 0.001) were observed in patients with normal nutritional status, whereas ferritin decreased significantly in patients with nutritional risk (*p* = 0.005). Given the exploratory nature of these analyses, these findings should be interpreted cautiously (Supplementary Table 4).

To determine whether nutritional risk was independently associated with functional outcome, multivariable linear regression was performed with discharge Barthel Index as the dependent variable (Table 5). In the primary adjusted model, which included baseline Barthel Index, nutritional risk group, age, sex, time from stroke onset to rehabilitation, stroke type, and baseline MMSE, nutritional risk remained significantly associated with a lower discharge Barthel Index (B = −2.99, 95% CI −5.65 to −0.33, *p* = 0.028). Baseline Barthel Index was the strongest predictor of discharge Barthel Index in this model. In the sensitivity model, which additionally included hypertension and baseline serum albumin, the association remained significant (B = −3.38, 95% CI –6.33 to −0.42, *p* = 0.026). Model diagnostics supported the adequacy of both regression models, with low multicollinearity, Durbin-Watson statistics close to 2, and no influential cases identified.

**Table 5:** Multivariable linear regression models examining the association between nutritional risk and Barthel Index at discharge.

Predictor	Primary adjusted model B (95% CI)	*p* value	Sensitivity model B (95% CI)	*p* value
Nutritional risk group (at risk vs. normal)	−2.99 (−5.65 to −0.33)	0.028	−3.38 (−6.33 to −0.42)	0.026
Baseline Barthel Index	1.04 (0.96 to 1.11)	<0.001	1.04 (0.96 to 1.13)	<0.001
Age (years)	0.02 (−0.11 to 0.14)	0.781	0.01 (−0.11 to 0.14)	0.846
Sex (male vs. female)	−0.79 (−3.42 to 1.84)	0.551	−0.74 (−3.40 to 1.92)	0.578
Time from stroke onset to rehabilitation (months)	−0.09 (−0.67 to 0.48)	0.746	−0.08 (−0.68 to 0.52)	0.799
Stroke type (hemorrhagic vs. ischemic)	−1.69 (−5.76 to 2.39)	0.410	−1.82 (−5.91 to 2.28)	0.377
Baseline MMSE	−0.48 (−0.99 to 0.03)	0.065	−0.45 (−0.97 to 0.06)	0.081
Hypertension (yes vs. no)	—	—	−0.30 (−3.21 to 2.61)	0.836
Baseline serum albumin (g/L)	—	—	−0.24 (−0.61 to 0.12)	0.184

Model fit: Primary model: R^2^ = 0.954, adjusted R^2^ = 0.947, RMSE = 4.62, F = 152.8, *n* = 60. Sensitivity model: R^2^ = 0.955, adjusted R^2^ = 0.947, RMSE = 4.63, F = 118.7, *n* = 60. CI, confidence interval; MMSE, Mini-Mental State Examination; B, unstandardized regression coefficient; RMSE, root mean square error; VIF, variance inflation factor.

Discharge Barthel Index was used as the dependent variable in all models. The primary adjusted model included nutritional risk group, baseline Barthel Index, age, sex, time from stroke onset to rehabilitation, stroke type, and baseline MMSE. The sensitivity model additionally included hypertension and baseline serum albumin. Unstandardized coefficients are presented to facilitate clinical interpretation. Negative coefficients indicate lower discharge Barthel Index values associated with the corresponding predictor. Both models were estimated using complete-case analysis. Regression diagnostics supported the adequacy of both models: in the primary model, variance inflation factors ranged from 1.039 to 1.610 and the Durbin–Watson statistic was 2.131 (*p* = 0.608), whereas in the sensitivity model, variance inflation factors ranged from 1.046 to 1.771 and the Durbin–Watson statistic was 2.139 (*p* = 0.608). No influential cases were identified in either model.

Secondary adjusted analyses were performed for discharge MMSE and for categorical improvement in FAC and mRS. In multivariable linear regression, nutritional risk was not independently associated with discharge MMSE, although the direction of the association remained negative in both the primary and sensitivity models. Likewise, in adjusted logistic regression analyses, nutritional risk was not significantly associated with the odds of FAC or mRS improvement. These secondary regression findings are presented in Supplementary Tables 2–4.

Nutritional status also changed significantly during the rehabilitation period. Most patients with normal nutritional status at baseline remained in the same category at discharge (36/38, 94.7%), whereas 9 of 22 patients initially classified as having nutritional risk were reclassified as having normal nutritional status after treatment. Overall, the cross-classified distribution of MNA-SF categories differed significantly between baseline and discharge assessments (χ^2^ = 21.53, df = 1, *p* < 0.001).

## Discussion

In this study, we evaluated the relationship between screening-defined nutritional risk, abdominal muscle thickness, and rehabilitation outcomes in patients with subacute to chronic stroke. The main finding was that nutritional risk was independently associated with lower functional independence at discharge, as measured by the Barthel Index. This association was limited to functional outcomes, as no independent relationship was observed with cognitive recovery (MMSE) or mobility outcomes (FAC and mRS) after adjustment for baseline variables. Although patients with normal nutritional status demonstrated higher MMSE scores at both baseline and post-treatment assessments, nutritional risk was not independently associated with cognitive outcomes in multivariable analyses. This suggests that the observed differences in cognitive performance may largely reflect baseline clinical characteristics rather than a direct effect of nutritional risk.

With regard to functional mobility, FAC scores did not differ significantly between groups at baseline or after rehabilitation. Similarly, both groups showed improvements in motor recovery following rehabilitation, as assessed by Brunnstrom stages. While patients with normal nutritional status tended to have higher baseline motor scores, these differences were not statistically significant, and both groups benefited from the rehabilitation program.

Barthel Index scores were consistently higher in patients with normal nutritional status at both baseline and post-treatment evaluations. Although patients with nutritional risk also demonstrated improvement over time, these changes did not consistently reach statistical significance. Together with the adjusted analyses, these findings suggest that nutritional risk may be associated with lower levels of functional independence, rather than preventing overall recovery.

Regarding abdominal muscle morphology, no significant differences were observed in muscle thickness either at baseline or following rehabilitation, and no association was found with nutritional status. These findings indicate that changes in functional outcomes were observed without measurable concurrent alterations in abdominal muscle thickness under the conditions of this study. However, this result should be interpreted with caution. Muscle assessment was limited to thickness measurements on the non-paretic side over a relatively short follow-up period, and did not include parameters reflecting muscle quality or neuromuscular activation. Therefore, no mechanistic conclusions can be drawn, and the findings should be considered descriptive.

In the literature, a higher muscle mass ratio and better nutritional status in stroke patients have been reported to be significantly associated with Functional Independence Measure (FIM) scores following rehabilitation (42). Moreover, improvements in nutritional status and diet quality have been shown to positively affect motor and cognitive functions, handgrip strength, and quality of life, contributing to reduced disability and improved activities of daily living (12–14, 43, 44). Poor nutritional status has been implicated in the development of post-stroke cognitive impairment, particularly affecting global cognitive function and frontal lobe–related processes (45). Previous studies have shown that better nutritional status has been associated with improved functional outcomes after stroke rehabilitation. However, our results suggest that this relationship may be more robust for functional independence than for cognitive or mobility outcomes when potential confounders are taken into account.

Serum albumin levels were significantly lower in patients with nutritional risk compared with those with normal nutritional status. The coexistence of low BMI and low serum albumin levels has previously been shown to be significantly associated with poor functional recovery in subacute stroke patients (46). In a retrospective study evaluating stroke rehabilitation outcomes, positive correlations were reported between albumin and hemoglobin levels and FAC scores (47). Similarly, another study demonstrated an association between serum albumin levels and limitations in activities of daily living (48). Although serum albumin is known to be related to malnutrition and Mini Nutritional Assessment scores, it is also influenced by multiple factors, including muscle mass, hypovolemia, comorbidities, increased catabolism, and inflammation (49). In our study, serum albumin levels remained relatively stable in patients with nutritional risk, whereas a significant decrease was observed in patients with normal nutritional status after treatment. Previous studies have reported that the recovery rate of plasma albumin levels following high-intensity exercise is slower in older adults compared with younger individuals. This has been attributed to age related reductions in hepatic albumin synthesis or insufficient protein intake to meet increased metabolic demands (50). In this context, the post-treatment decline in albumin levels observed in patients with normal nutritional status in our study may reflect the increased metabolic demands associated with the rehabilitation process rather than a true deterioration in nutritional status. This finding should be interpreted with caution, as serum albumin is influenced by multiple non-nutritional factors, including inflammation and comorbid conditions. In addition, baseline Prognostic Nutritional Index (PNI) values were, as expected, significantly higher in patients with normal nutritional status than in those with nutritional risk. The post-treatment decrease in PNI observed in patients with normal nutritional status may be primarily driven by changes in serum albumin levels rather than reflecting a true deterioration in nutritional status.

A longitudinal study evaluating the long-term course of abdominal muscles in patients with chronic stroke reported no significant changes in the thickness of the internal oblique, external oblique, or transversus abdominis muscles over a two-year follow-up period. In the same study, echo intensity analysis showed no significant change on the non-paretic side, whereas an increase in echo intensity was observed only in the external oblique muscle on the paretic side (26). Another study comparing abdominal muscles between stroke patients and healthy adults found greater rectus abdominis thickness on the non-paretic side in stroke patients than in healthy controls (27). In another study examining the relationship between trunk muscles and functional outcomes in stroke patients, posterior trunk muscles, particularly quadratus lumborum thickness, were associated with balance and FAC scores. In contrast, no significant associations were found between functional parameters and the internal oblique, external oblique, or transversus abdominis muscles (29). In a study comparing standard rehabilitation alone with standard rehabilitation combined with core stabilization exercises, no significant between group differences were observed in external or internal oblique muscle thickness on the paretic side at the end of treatment. However, a significant increase in transversus abdominis thickness was detected only in the group receiving core stabilization exercises (51). Similarly, another study comparing paretic and non-paretic sides in stroke patients found no significant differences in abdominal muscle thickness or echo intensity values between sides (25). In our study, no significant association was found between abdominal muscle thickness and functional outcomes or nutritional status. However, these findings should be interpreted with caution. Given that our assessment was limited to muscle thickness on the non-paretic side over a relatively short 4-week period, the data do not support firm mechanistic conclusions. Within these constraints, abdominal muscle thickness alone may not be a sufficiently sensitive marker to reflect functional recovery.

### Limitations

This study has several limitations. First, ultrasonographic assessments did not include echo intensity analysis, which reflects muscle quality; instead, the evaluation focused solely on muscle thickness, which may be considered a limitation. In addition, muscle thickness measurements were performed unilaterally, precluding bilateral comparisons. Nevertheless, performing measurements on the non-paretic side was intended to minimize measurement bias related to increased tone and spasticity. Another limitation is that patients’ nutritional status was classified based solely on MNA-SF as a screening tool, and a comprehensive diagnostic evaluation using GLIM criteria or additional biochemical and dietary assessments could not be performed. In addition, rehabilitation dose and exercise intensity were not objectively quantified; therefore, differences in treatment engagement between groups cannot be excluded and may have influenced the outcomes. The relatively short duration of the rehabilitation program (4 weeks) may have limited the ability to detect measurable changes in muscle thickness. Furthermore, the single center design of the study and the relatively small sample size may limit the generalizability of the findings.

## Conclusion

This study suggests that functional improvement was observed without measurable concurrent changes in abdominal muscle thickness under the present assessment approach. Although abdominal muscle thickness remained unchanged, the association between nutritional risk and functional independence indicates that nutritional, biochemical, and functional parameters should be considered together in clinical evaluation. These findings highlight the importance of early and comprehensive nutritional assessment during the rehabilitation process. Future studies with larger sample sizes, multicenter designs, and advanced ultrasonographic evaluations incorporating muscle quality measures are warranted to more comprehensively elucidate the relationship between nutritional status, muscle morphology, and functional outcomes in patients with stroke.

## Data Availability

The data analyzed in this study is subject to the following licenses/restrictions: the dataset contains sensitive patient information and is therefore not publicly available due to ethical and confidentiality restrictions, but may be made available from the corresponding author upon reasonable request. Requests to access these datasets should be directed to Esra Şahingöz Bakırcı, dresrasahingoz@gmail.com.
