# Supplementary material for: Nutritional status, abdominal muscle thickness, and functional outcomes in patients with subacute to chronic stroke
Source: Front Neurol. 2026 Apr 28;17:1794367. doi: 10.3389/fneur.2026.1794367 (PMC13160780; doi:10.3389/fneur.2026.1794367)
Supplement: Supplementary file 1 [file Table_1.DOCX]

**Supplementary Table 1. Detailed pre–post shift analyses and within-group changes in functional, cognitive, ultrasound, and laboratory outcomes according to nutritional risk group**

|  |  | Patients with normal nutritional status | | | | Patients with nutritional risk | | | |  |  |
| --- | --- | --- | --- | --- | --- | --- | --- | --- | --- | --- | --- |
| Variables | Subcategory | Baseline | Post-treatment | **Shift Analysis** | p value (within) | Baseline | Post-treatment |  | p value (within) | p value (between post-treatment) | p value (shift groups) |
| BMRS – Upper extremity |  | 4 (3-5) | 5 (4-6) |  | <0.001 | 3 (3-4) | 4 (3-4.8) |  | <0.001 | 0.017 | 0.710 |
|  | 1 | 2 (5.3%) | 0 (0%) | **unchanged [12 (31.6%)]** |  | 1 (4.5%) | 0 (0%) | **unchanged [8 (36.4%)]** |  |  |  |
|  | 2 | 3 (7.9%) | 3 (7.9%) | **1-stage improvement [25 (65.8%)]** |  | 3 (13.6%) | 2 (9.1%) | **1-stage improvement [14 (63.6%)]** |  |  |  |
|  | 3 | 8 (21.1%) | 3 (7.9%) | **2-stage improvement [1 (2.6%)]** |  | 10 (45.5%) | 6 (27.3%) | **2-stage improvement [0 (0%)]** |  |  |  |
|  | 4 | 7 (18.4%) | 8 (21.1%) |  |  | 6 (27.3%) | 8 (36.4%) |  |  |  |  |
|  | 5 | 17 (44.7%) | 13 (34.2%) |  |  | 1 (4.5%) | 4 (18.2%) |  |  |  |  |
|  | 6 | 1 (2.6%) | 11 (28.9%) |  |  | 1 (4.5%) | 2 (9.1%) |  |  |  |  |
| BMRS - Hand |  | 4 (3-5) | 5 (4-6) |  | <0.001 | 3 (2-4) | 4 (3-5) |  | <0.001 | 0.029 | 0.455 |
|  | 1 | 4 (10.5%) | 3 (7.9%) | **1-level worsening [0 (0%)]** |  | 3 (13.6%) | 2 (9.1%) | **1-level worsening [1 (4.6%)]** |  |  |  |
|  | 2 | 4 (10.5%) | 1 (2.6%) | **unchanged [7 (18.4%)]** |  | 5 (22.7%) | 1 (4.5%) | **unchanged [6 (27.3%)]** |  |  |  |
|  | 3 | 6 (15.8%) | 3 (7.9%) | **1-level improvement [26 (68.4%)]** |  | 6 (27.3%) | 6 (27.3%) | **1-level improvement [13 (59.1%)]** |  |  |  |
|  | 4 | 9 (23.7%) | 7 (18.4%) | **2-level improvement [5 (13.2%)]** |  | 5 (22.7%) | 6 (27.3%) | **2-level improvement [2 (9.1%)]** |  |  |  |
|  | 5 | 15 (39.5%) | 9 (23.7%) |  |  | 2 (9.1%) | 5 (22.7%) |  |  |  |  |
|  | 6 | 0 (0%) | 15 (39.5%) |  |  | 1 (4.5%) | 2 (9.1%) |  |  |  |  |
| BMRS – Lower extremity |  | 5 (4-5) | 5 (5-6) |  | <0.001 | 4 (3-4) | 4 (4-5) |  | <0.001 | 0.003 | 0.630 |
|  | 1 | 0 (0%) | 0 (0%) | **unchanged [14 (36.8%)]** |  | 0 (0%) | 0 (0%) | **unchanged [10 (45.5%)]** |  |  |  |
|  | 2 | 1 (2.6%) | 0 (0%) | **1-stage improvement [23 (60.5%)]** |  | 0 (0%) | 0 (0%) | **1-stage improvement [12 (54.5%)]** |  |  |  |
|  | 3 | 5 (13.2%) | 3 (7.9%) | **2-stage improvement [1 (2.6%)]** |  | 9 (40.9%) | 4 (18.2%) | **2-stage improvement [0 (0%)]** |  |  |  |
|  | 4 | 11 (28.9%) | 5 (13.2%) |  |  | 9 (40.9%) | 9 (40.9%) |  |  |  |  |
|  | 5 | 20 (52.6%) | 17 (44.7%) |  |  | 4 (18.2%) | 7 (31.8%) |  |  |  |  |
|  | 6 | 1 (2.6%) | 13 (34.2%) |  |  | 0 (0%) | 2 (9.1%) |  |  |  |  |
| FAC |  | 4 (2-4) | 4 (2-5) |  | 0.036 | 2 (1-4) | 2.5 (1-4.8) |  | 0.020 | 0.184 | 0.379 |
|  | 0 | 0 (0%) | 1 (2.6%) | **1-level worsening [3 (7.9%)]** |  | 2 (9.1%) | 1 (4.5%) | **1-level worsening [0 (0%)]** |  |  |  |
|  | 1 | 7 (18.4%) | 3 (7.9%) | **unchanged [24 (63.2%)]** |  | 5 (22.7%) | 6 (27.3%) | **unchanged [16 (72.7%)]** |  |  |  |
|  | 2 | 9 (23.7%) | 10 (26.3%) | **1-level improvement [11 (28.9%)]** |  | 5 (22.7%) | 4 (18.2%) | **1-level improvement [6 (27.3%)]** |  |  |  |
|  | 3 | 1 (2.6%) | 3 (7.9%) |  |  | 2 (9.1%) | 2 (9.1%) |  |  |  |  |
|  | 4 | 12 (31.6%) | 8 (21.1%) |  |  | 5 (22.7%) | 3 (13.6%) |  |  |  |  |
|  | 5 | 9 (23.7%) | 13 (34.2%) |  |  | 3 (13.6%) | 6 (27.3%) |  |  |  |  |
| mRS |  | 2 (1-4) | 2 (1-3.8) |  | 0.036 | 3 (2-4) | 3 (1.3-4) |  | 0.393 | 0.099 | 0.424 |
|  | 0 | 2 (5.3%) | 6 (15.8%) | **1-point improvement [11 (28.9%)]** |  | 0 (0%) | 3 (13.6%) | **1-point improvement [7 (31.8%)]** |  |  |  |
|  | 1 | 16 (42.1%) | 10 (26.3%) | **unchanged [24 (63.2%)]** |  | 5 (22.7%) | 3 (13.6%) | **unchanged [11 (50%)]** |  |  |  |
|  | 2 | 2 (5.3%) | 7 (18.4%) | **1-point worsening [3 (7.9%)]** |  | 3 (13.6%) | 3 (13.6%) | **1-point worsening [4 (18.2%)]** |  |  |  |
|  | 3 | 5 (13.2%) | 5 (13.2%) |  |  | 4 (18.2%) | 3 (13.6%) |  |  |  |  |
|  | 4 | 13 (34.2%) | 10 (26.3%) |  |  | 9 (40.9%) | 7 (31.8%) |  |  |  |  |
|  | 5 | 0 (0%) | 0 (0%) |  |  | 1 (4.5%) | 3 (13.6%) |  |  |  |  |
| Barthel Index |  | 82.5 (65-90) | 87.5 (66.3-95) |  | <0.001 | 65 (51.3-85) | 67.5 (60-88.8) |  | 0.053 | 0.008 |  |
| MMSE |  | 25 (22.5-26) | 26 (24-27.8) |  | 0.005 | 22 (22-24.8) | 23.5 (20.3-26) |  | >0.999 | 0.007 | 0.081 |
|  |  |  |  | **<-1 skor change [7 (18.4%)]** |  |  |  | **<-1 skor change [10 (45.5%)]** |  |  |  |
|  |  |  |  | **0 to 1 skor change [13 (34.2%)]** |  |  |  | **0 to 1 skor change [5 (22.7%)]** |  |  |  |
|  |  |  |  | **>1 skor increase [18 (47.4%)]** |  |  |  | **>1 skor increase [7 (31.8%)]** |  |  |  |
| EO muscle thickness (cm) |  | 3.4 (2.9-4.4) | 3.3 (2.9-4.1) |  | 0.350 | 3.5 (2.8-4.6) | 3.3 (2.8-4.4) |  | 0.346 | 0.616 |  |
| IO muscle thickness (cm) |  | 5.4 (4.5-6.7) | 5.1 (4.3-6.5) |  | 0.087 | 5.6 (4.4-6.7) | 5.9 (4.2-7.1) |  | 0.721 | 0.436 |  |
| TrA muscle thickness (cm) |  | 3.3 (2.8-4.4) | 3.5 (2.8-4.2) |  | 0.706 | 3.1 (2.7-4.3) | 3.4 (2.8-4.5) |  | 0.871 | 0.301 |  |
| WBC count (10³/µL) |  | 7.4 (6.5-8.9) | 7.6 (5.8-8.5) |  | 0.116 | 8.1 (6.9-8.8) | 8.5 (6.9-9.3) |  | 0.527 | 0.134 |  |
| Platelet count (10³/µL) |  | 257 (209-300) | 249.5 (225.8-303.8) |  | 0.733 | 235.5 (186.8-317.8) | 211.5 (174-273) |  | 0.020 | 0.05 |  |
| Neutrophil count (10³/µL) |  | 4.8 (3.7-5.2) | 4.6 (3-5.3) |  | 0.035 | 5 (4.2-5.9) | 5.2 (4.2-5.6) |  | 0.321 | 0.202 |  |
| Lymphocyte count (10³/µL) |  | 2.1 (1.6-2.7) | 2.2 (1.7-2.6) |  | 0.827 | 2 (1.6-2.6) | 2.1 (1.6-2.6) |  | 0.121 | 0.346 |  |
| C-reactive protein (mg/L) |  | 4.3 (2-7.8) | 4.6 (3.1-8.2) |  | 0.959 | 3.8 (2-6.8) | 3.2 (2-7.1) |  | 0.520 | 0.170 |  |
| Serum albumin (g/L) |  | 44.7 (42.1-45.9) | 41 (38.3-43.2) |  | <0.001 | 41.3 (40-44.1) | 40.8 (39.1-42.8) |  | 0.467 | 0.704 |  |
| Serum ferritin (ng/mL) |  | 68.7 (36.4-116.6) | 57.7 (37.9-105.2) |  | 0.632 | 90 (36.5-171) | 68.2 (24.2-120.5) |  | 0.005 | 0.923 |  |
| Total protein (g/L) |  | 69.5 (65.3-74.9) | 68.9 (65.8-71.9) |  | 0.131 | 69.7 (66-72.8) | 70.6 (65.7-73.9) |  | 0.697 | 0.919 |  |
| PNI |  | 54.2 (50.6-58.9) | 50.7 (47.4-55.6) |  | <0.001 | 51 (47.7-55.8) | 51.7 (50.4-54.3) |  | 0.372 | 0.359 |  |

Abbreviations: SMD: standard mean difference, BMRS: Brunnstrom Motor Recovery Stages, FAC: Functional Ambulation Category, mRS: Modified Rankin Scale, MMSE: Mini-Mental State Examination, EO: External oblique, IO: Internal oblique, TrA: Transversus abdominis, WBC: White blood cell, PNI: prognostic nutritional index. Within-group p values were calculated using the Wilcoxon signed-rank test. Shift categories are presented descriptively as frequencies and percentages. Between-group comparisons of post-treatment values were performed using the Mann–Whitney U test, and between-group comparisons of shift distributions were assessed using the chi-square test, as appropriate.

**Supplementary Table 2. Multivariable linear regression analyses for discharge MMSE**

| Predictor | Primary adjusted model B (95% CI) | p value | Sensitivity model B (95% CI) | p value |
| --- | --- | --- | --- | --- |
| Patients with nutritional risk (at risk vs normal) | -0.92 (-2.01 to 0.18) | 0.101 | -1.15 (-2.38 to 0.09) | 0.067 |
| Baseline MMSE | 0.75 (0.54 to 0.96) | <0.001 | 0.75 (0.54 to 0.96) | <0.001 |
| Age (years) | -0.06 (-0.11 to -0.01) | 0.027 | -0.06 (-0.11 to -0.01) | 0.023 |
| Sex (male vs female) | 0.07 (-1.02 to 1.16) | 0.898 | 0.13 (-0.98 to 1.24) | 0.818 |
| Time from stroke onset to rehabilitation (months) | 0.13 (-0.11 to 0.36) | 0.295 | 0.10 (-0.15 to 0.35) | 0.412 |
| Stroke type (hemorrhagic vs ischemic) | 0.07 (-1.61 to 1.75) | 0.93 | 0.01 (-1.69 to 1.72) | 0.986 |
| Baseline Barthel Index | 0.02 (-0.01 to 0.05) | 0.204 | 0.02 (-0.02 to 0.05) | 0.267 |
| Hypertension (yes vs no) | — | — | 0.37 (-0.84 to 1.59) | 0.538 |
| Baseline serum albumin (g/L) | — | — | -0.04 (-0.19 to 0.11) | 0.616 |
| Model fit: Primary model: R² = 0.722, adjusted R² = 0.685, RMSE = 1.91, F = 19.32, n = 60. Sensitivity model: R² = 0.727, adjusted R² = 0.677, RMSE = 1.93, F = 14.77, n = 60. | | | | |

Abbreviations: MMSE, Mini-Mental State Examination; CI, confidence interval; B, unstandardized regression coefficient; RMSE, root mean square error; VIF, variance inflation factor. Discharge MMSE was used as the dependent variable. The primary adjusted model included nutritional risk group, baseline MMSE, age, sex, time from stroke onset to rehabilitation, stroke type, and baseline Barthel Index. The sensitivity model additionally included hypertension and baseline serum albumin. Unstandardized coefficients are presented with 95% confidence intervals. Regression diagnostics supported the adequacy of both models: in the primary model, variance inflation factors ranged from 1.039 to 1.610 and the Durbin–Watson statistic was 2.250 (p = 0.327), whereas in the sensitivity model, variance inflation factors ranged from 1.046 to 1.771 and the Durbin–Watson statistic was 2.148 (p = 0.581). No influential cases were identified in either model.

**Supplementary Table 3. Adjusted logistic regression analyses for improvement in FAC**

| Predictor | Primary adjusted model OR (95% CI) | p value | Sensitivity model OR (95% CI) | p value |
| --- | --- | --- | --- | --- |
| Patients with nutritional risk (at risk vs normal) | 0.66 (0.16 to 2.83) | 0.580 | 0.66 (0.15 to 2.97) | 0.590 |
| Baseline FAC | 0.86 (0.56 to 1.31) | 0.476 | 0.84 (0.55 to 1.28) | 0.424 |
| Baseline MMSE | 0.91 (0.71 to 1.16) | 0.429 | 0.90 (0.71 to 1.15) | 0.411 |
| Age (years) | 0.95 (0.89 to 1.01) | 0.124 | 0.95 (0.89 to 1.02) | 0.125 |
| Sex (male vs female) | 1.16 (0.32 to 4.23) | 0.82 | 1.17 (0.31 to 4.40) | 0.815 |
| Time from stroke onset to rehabilitation (months) | 0.91 (0.67 to 1.23) | 0.53 | 0.90 (0.66 to 1.22) | 0.479 |
| Hypertension (yes vs no) | — | — | 1.20 (0.33 to 4.34) | 0.786 |
| Baseline serum albumin (g/L) | — | — | 1.02 (0.86 to 1.22) | 0.792 |
| Model fit: Primary model: McFadden R² = 0.112, Nagelkerke R² = 0.179, AUC = 0.724, n = 60. Sensitivity model: McFadden R² = 0.113, Nagelkerke R² = 0.182, AUC = 0.726, n = 60. | | | | |

Abbreviations: FAC, Functional Ambulation Category; OR, odds ratio; CI, confidence interval; AUC, area under the receiver operating characteristic curve; VIF, variance inflation factor. Binary logistic regression was used to examine improvement in FAC, defined as at least one-category increase from baseline. The primary adjusted model included nutritional risk group, baseline FAC, baseline MMSE, age, sex, and time from stroke onset to rehabilitation. The sensitivity model additionally included hypertension and baseline serum albumin. Odds ratios with 95% confidence intervals are presented. Model diagnostics were acceptable in both analyses, with variance inflation factors ranging from 1.000 to 1.358 in the primary model and from 1.000 to 1.499 in the sensitivity model. No influential cases were identified in either model. Model discrimination was modest, with an AUC of 0.724 in the primary model and 0.726 in the sensitivity model. Note: Stroke type was excluded from the multivariate models due to the small size of the hemorrhagic subgroup (n=6) to maintain model stability.

**Supplementary Table 4. Adjusted logistic regression analyses for improvement in mRS**

| Predictor | Primary adjusted model OR (95% CI) | p value | Sensitivity model OR (95% CI) | p value |
| --- | --- | --- | --- | --- |
| Patients with nutritional risk (at risk vs normal) | 1.77 (0.45 to 7.05) | 0.418 | 2.14 (0.41 to 11.14) | 0.365 |
| Baseline mRS | 1.25 (0.76 to 2.05) | 0.38 | 1.14 (0.69 to 1.88) | 0.603 |
| Baseline MMSE | 1.15 (0.92 to 1.45) | 0.211 | 1.19 (0.92 to 1.54) | 0.194 |
| Age (years) | 1.03 (0.97 to 1.10) | 0.303 | 1.04 (0.97 to 1.00*) | 0.266 |
| Sex (male vs female) | 6.57 (1.29 to 33.55) | 0.024 | 6.03 (1.26 to 28.92) | 0.025 |
| Time from stroke onset to rehabilitation (months) | 1.28 (0.99 to 1.64) | 0.056 | 1.35 (0.99 to 1.85) | 0.059 |
| Hypertension (yes vs no) | — | — | 0.40 (0.08 to 2.06) | 0.272 |
| Baseline serum albumin (g/L) | — | — | 0.94 (0.76 to 1.17) | 0.600 |
| Model fit: Primary model: McFadden R² = 0.190, Nagelkerke R² = 0.294, AUC = 0.771, n = 60. Sensitivity model: McFadden R² = 0.212, Nagelkerke R² = 0.323, AUC = 0.799, n = 60. | | | | |

Abbreviations: mRS, modified Rankin Scale; OR, odds ratio; CI, confidence interval; AUC, area under the receiver operating characteristic curve; VIF, variance inflation factor. Binary logistic regression was used to examine improvement in mRS, defined as at least one-category decrease from baseline. The primary adjusted model included nutritional risk group, baseline mRS, baseline MMSE, age, sex, and time from stroke onset to rehabilitation. The sensitivity model additionally included hypertension and baseline serum albumin. Odds ratios with 95% confidence intervals are presented. Model diagnostics were acceptable in both analyses, with variance inflation factors ranging from 1.000 to 1.509 in the primary model and from 1.000 to 1.551 in the sensitivity model. No influential cases were identified in either model. Model discrimination was acceptable, with an AUC of 0.771 in the primary model and 0.799 in the sensitivity model. Note: Stroke type was excluded from the multivariate models due to the small size of the hemorrhagic subgroup (n=6) to maintain model stability.

**Supplementary Figure 1. Love plot of standardized mean differences for baseline variables**
